# Correction: Unique post-translational oxime formation in the biosynthesis of the azolemycin complex of novel ribosomal peptides from *Streptomyces* sp. FXJ1.264

**DOI:** 10.1039/c5sc90063h

**Published:** 2015-11-05

**Authors:** Ning Liu, Lijiang Song, Minghao Liu, Fei Shang, Zoe Anderson, David J. Fox, Gregory L. Challis, Ying Huang

**Affiliations:** a State Key Laboratory of Microbial Resources, Institute of Microbiology, Chinese Academy of Sciences, Beijing 100101, P. R. China. Email: huangy@im.ac.cn; Fax: +86 10 64807311; Tel: +86 10 64807311; b Department of Chemistry, University of Warwick, Coventry, UK CV4 7AL. Email: G.L.Challis@warwick.ac.uk; Fax: +44 (0)2476 524112; Tel: +44 (0)2476 574024; c University of Chinese Academy of Sciences, Beijing, 100049, P. R. China; d Analytical and Testing Center, Beijing University of Chemical Technology, Beijing 100029, P. R. China

## Abstract

Correction for ‘Unique post-translational oxime formation in the biosynthesis of the azolemycin complex of novel ribosomal peptides from *Streptomyces* sp. FXJ1.264’ by Ning Liu *et al.*, *Chem. Sci.*, 2015, DOI: 10.1039/c5sc03021h.



## 

In the Conclusions section of the manuscript, the species of *Streptomyces* is displayed incorrectly; it should be “*Streptomyces* sp. FXJ1.264” rather than “*Streptomyces* sp. FXJ1.261”.

The Royal Society of Chemistry apologises for these errors and any consequent inconvenience to authors and readers.

